# Loss of the Heparan Sulfate Sulfotransferase, *Ndst1*, in Mammary Epithelial Cells Selectively Blocks Lobuloalveolar Development in Mice

**DOI:** 10.1371/journal.pone.0010691

**Published:** 2010-05-18

**Authors:** Brett E. Crawford, Omai B. Garner, Joseph R. Bishop, David Y. Zhang, Kevin T. Bush, Sanjay K. Nigam, Jeffrey D. Esko

**Affiliations:** 1 Department of Cellular and Molecular Medicine, University of California San Diego, La Jolla, California, United States of America; 2 Department of Pediatrics, University of California San Diego, La Jolla, California, United States of America; 3 Department of Medicine, University of California San Diego, La Jolla, California, United States of America; 4 Biomedical Sciences Graduate Program, University of California San Diego, La Jolla, California, United States of America; Katholieke Universiteit Leuven, Belgium

## Abstract

**Background:**

Considerable evidence indicates that heparan sulfate is essential for the development of tissues consisting of branching ducts and tubules. However, there are few examples where specific sulfate residues regulate a specific stage in the formation of such tissues.

**Methodology/Principal Findings:**

We examined the role of heparan sulfation in mammary gland branching morphogenesis, lactation and lobuloalveolar development by inactivation of heparan sulfate GlcNAc N-deacetylase/N-sulfotransferase genes (*Ndst*) in mammary epithelial cells using the Cre-loxP system. *Ndst1* deficiency resulted in an overall reduction in glucosamine N-sulfation and decreased binding of FGF to mammary epithelial cells *in vitro* and *in vivo*. Mammary epithelia lacking *Ndst1* underwent branching morphogenesis, filling the gland with ductal tissue by sexual maturity to the same extent as wildtype epithelia. However, lobuloalveolar expansion did not occur in *Ndst1*-deficient animals, resulting in insufficient milk production to nurture newly born pups. Lactational differentiation of isolated mammary epithelial cells occurred appropriately via stat5 activation, further supporting the notion that the lack of milk production was due to lack of expansion of the lobuloalveoli.

**Conclusions/Significance:**

These findings demonstrate a selective, highly penetrant, cell autonomous effect of *Ndst1*-mediated sulfation on lobuloalveolar development.

## Introduction

Mammary gland development occurs in multiple stages: (i) fetal development of the rudimentary mammary buds, (ii) branching morphogenesis in immature animals, (iii) formation of lobular alveoli during pregnancy, (iv) differentiation of milk-producing epithelia and lactation, and (v) involution after weaning. The classical endocrine hormones (estrogen, progesterone, growth hormone, and prolactin) regulate these developmental processes by acting on the mammary stroma to induce the local expression of soluble growth factors [1].

Based on genetic studies, a large family of growth factors regulate these processes, including fibroblast growth factors (FGFs), Wnts, parathyroid hormone-related protein (PTHrP), Hedgehog proteins (Hh and Ihh), transforming growth factor beta (TGFβ) and inhibin-βb, insulin-like growth factor(IGF)-I and -II and IGF-binding protein-5 (IGFBP-5), hepatocyte growth factor (HGF), amphiregulin, epidermal growth factor (EGF) and EGF receptors, and heregulin (HRG) and ERBB4 receptors [2], [3], [4], [5], [6], [7], [8], [9], [10], [11], [12], [13], [14], [15], [16], [17], [18], [19], [20], [21]. Nearly all of these factors bind to heparan sulfate, a glycosaminoglycan present in extracellular matrix and cell surface proteoglycans. Interaction with heparan sulfate is hypothesized to protect growth factors against degradation, to create a storage depot for later release, to facilitate assembly of signaling complexes (co-receptor activity), to enable clearance by endocytosis, and to regulate growth factor diffusion through the tissue [22], [23], [24]. Heparan sulfate also can act indirectly in the system, e.g. by modulating the processing of growth factor precursors by matrix metalloproteases [25].

Heparan sulfate was one of the first extracellular matrix components identified in the developing mammary gland. Midpregnant mice produce glycosaminoglycans (GAGs) in the basal lamina and the surrounding matrix, including heparan sulfate and hyaluronan. GAG production appeared to be dynamically regulated, as the overall level of GAG declined at the end buds during puberty and during pregnancy [26]. Sulfated GAGs were found in the posterior (neck) region of the terminal end bud in the epithelia, stroma and elsewhere [27]. Further evidence supporting a role for heparan sulfate in mammary development derives from studies of genetically altered mouse strains. For example, hyperbranching occurs in the mammary epithelia of transgenic mice expressing human heparanase, a degradative enzyme secreted by cells [28]. Mice deficient in syndecan-1, a cell surface heparan sulfate proteoglycan expressed by mammary epithelial cells, exhibit normal primary mammary duct formation, but have a mild reduction in secondary and tertiary branching [29], [30]. In *CD44* ^−/−^ mice, postpartum uterine involution is accelerated and maintenance of lactation is impaired, presumably due to mislocalization of matrix metalloproteinase 7 and altered processing of proHB-EGF [31]. In some cases, the mechanism responsible for these developmental alterations has not been characterized, and one cannot discriminate cell-autonomous effects in the mammary epithelia versus stromal effects since the mutations were systemic. Mutants lacking other heparan sulfate proteoglycans either succumb embryonically [32], obviating further studies of the mammary gland, or have no reported defects in mammary gland development or function [33], [34], [35], [36], [37].

In general, the essential nature of heparan sulfate has made it difficult to study the effect of altering its biosynthesis in the mammary gland in vivo. Systemic deletion of *Ext1* or *Ext2* (subunits of the heparan sulfate copolymerase) results in early embryonic death (E6-7) due to failure to form mesoderm during gastrulation [38], [39]. Deletion of the gene encoding heparan sulfate GlcNAc N-deacetylase/N-sulfotransferase-1 (*Ndst1*), one of a family of four enzymes involved in the initial sulfation of the heparan sulfate chains, leads to perinatal lethality with lung, brain and skeletal defects [40], [41], [42]. Similarly, deletion of two other modifying enzymes, uronyl 2-O-sulfotransferase and the glucuronyl C5 epimerase, causes perinatal death due to kidney agenesis [43], [44], [45], [46]. In other organs, such as kidney, lung and salivary gland, defects in heparan sulfation have mainly been shown to affect branching morphogenesis [47], although the mechanism may not be as straight-forward as initially believed [48]. However, most of this work has been done using in vitro systems.

To study the in vivo role of heparan sulfate in the mammary gland, we used the Cre-*loxP* recombination system to delete *Ndst1* in a tissue-specific manner in mammary epithelia. Somewhat surprisingly, we found that inactivation of *Ndst1* does not affect branching morphogenesis or lactational differentiation, but instead causes a striking defect in lobuloalveolar expansion leading to insufficient milk production for survival of offspring.

## Results

### Ndst expression and targeting

**Figure 1:**
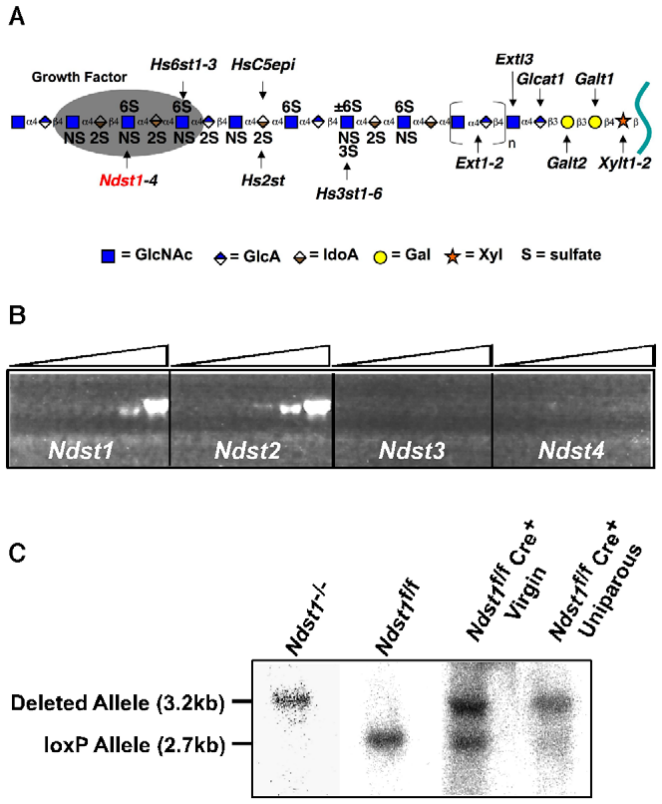
Inactivation of *Ndst1* in mammary epithelia. (**A**) Schematic diagram of heparan sulfate synthesis. The target gene, Ndst1, is shown in red. (**B**) RT-PCR analysis of Ndst expression in isolated epithelial cells. The ramp at top indicates increasing number of PCR amplification cycles. (**C**) Mammary epithelial cells were isolated from a virgin (lane 3) and a 3-month old uniparous female (lane 4) and analyzed by Southern blotting. For comparison, blots were performed on samples obtained from *Ndst1* deficient (lane 1) and *Ndst1* ^f/f^ (lane 2) mammary tumor cell lines (Experimental Procedures). *Ndst1* deletion was approximately 60% complete in adult virgin mice and increased to ∼80% after one pregnancy.

Heparan sulfate assembly occurs by copolymerization of N-acetylglucosamine (GlcNAc) and glucuronic acid (GlcA) residues, followed by a series of modification reactions in which segments of the chain undergo various sulfation reactions and a portion of the GlcA units epimerize to iduronic acid [49] (Fig. 1A). N-deacetylation and N-sulfation of subsets of the GlcNAc residues represents the first committed step in modifying the chains and the other modifications depend on this reaction. A family of four dual function enzymes, designated GlcNAc N-deacetylase/N-sulfotransferases (Ndsts) exists in vertebrates [50]. RT-PCR analysis showed that mammary epithelia express *Ndst1* and *Ndst2* but negligible levels of *Ndst3* and *Ndst4* (Fig. 1B). To determine the effects of altering the extent of heparan sulfation on mammary gland development, deletion mutants of *Ndst1* and *Ndst2* were examined.

*Ndst2* null animals lack connective tissue-type mast cells but are otherwise normal [51], [52]. In contrast, mice lacking *Ndst1* die perinatally due to lung insufficiency, as well as displaying skeletal and forebrain defects [40], [41], [42]. Thus, to examine the participation of *Ndst1* in the mammary gland, *Ndst1* was inactivated selectively in mammary epithelial cells by cross breeding mice bearing a floxed conditional allele (*Ndst1* ^f/f^) to *MMTVCre* mice, which express the bacteriophage Cre recombinase in mammary epithelia at day 6 postpartum [53], [54]. These mouse lines were interbred to generate *Ndst1* ^f/f^ *MMTVCre* ^+^ (mutant) and *Ndst1* ^f/f^ *MMTVCre* ^−^ (wildtype) mice. Both genotypes were obtained at the expected Mendelian frequency and appeared grossly normal.

Primary mammary epithelial cells were isolated from *Ndst1* ^f/f^ *MMTVCre* ^+^ and *Ndst1* ^f/f^ *MMTVCre* ^−^ virgin and uniparous female mice and expanded in tissue culture. Evaluation of markers for epithelial keratin and milk proteins indicated >90% purity. DNA isolated from the cells was analyzed by HindIII/BamHI digestion and Southern blotting. The wildtype “floxed” *Ndst1* allele generated a 2.7 kb band, whereas the deleted allele yielded a 3.2 kb band, based on analysis of clonal mammary epithelial cell lines derived from *Ndst1* ^f/f^ before and after Cre transfection in vitro (Fig. **1C**). The relative intensities of the two bands indicated that deletion of *Ndst1* in *Ndst1* ^f/f^ *MMTVCre* ^+^ was as high as ∼80% in multiparous females (Fig. **1C**), suggesting that some cells escaped recombination, possibly due to inefficient expression of Cre. Unless otherwise indicated, multiparous females were used in the in vivo studies that follow. In some work with isolated cells, >95% deletion was achieved by inactivating the gene use adenoviral Cre.

### *Ndst1* deficiency causes undersulfation of heparan sulfate in mammary ducts

**Figure 2:**
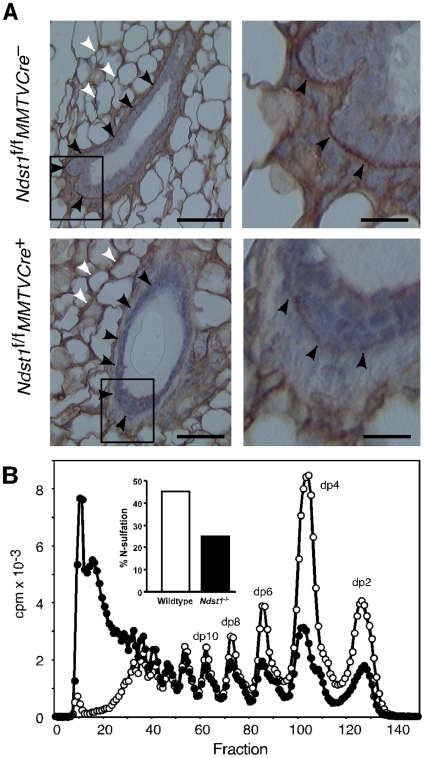
Altered expression of heparan sulfate in *Ndst1* ^f/f^ *MMTVCre* ^+^ mammary epithelia. (**A**) Frozen sections of mammary glands were incubated with biotinylated FGF2, which binds to heparan sulfate [55]. Binding of FGF2 was detected with streptavidin-HRP (brown stain). In wildtype *Ndst1* ^f/f^ *MMTVCre* ^–^ glands FGF2 binds to the basement membrane surrounding the epithelial ducts (arrowheads). The right panel magnifies the boxed region, revealing the sharp staining of the basement membrane underlying the epithelial cells. FGF2 also binds to the matrix surrounding the fat pad adipocytes (white arrowheads). Mutant *Ndst1* ^f/f^ *MMTVCre* ^+^ glands retain FGF2 binding around fat pad adipocytes, but binding to the basement membrane was greatly reduced. The lower right panel magnifies the boxed region. The bar in the left panels  = 50 µm, right panels 12.5 µm. (**B**) Heparan sulfate was isolated from [6-^3^H]glucosamine labeled mammary epithelial cells derived from *Ndst1* ^f/f^ *MMTVCre* ^–^ and *Ndst1* ^f/f^ *MMTVCre* ^+^ mice and degraded with nitrous acid [58]. The individual oligosaccharides were separated by gel filtration chromatography and the area under the peaks was used to determine the extent of N-sulfation of the chains [59]. dp, degree of polymerization. Inset: Graph of comparison of areas under the curves.

To determine how *Ndst1* deficiency affected heparan sulfate of mammary epithelia, frozen sections of *Ndst1* ^f/f^ *MMTVCre* ^−^ and *Ndst1* ^f/f^ *MMTVCre* ^+^ mammary glands were incubated with a biotinylated form of basic fibroblast growth factor (FGF2) and then stained with streptavidin-horse radish peroxidase [55]. FGF2 binds with high affinity to heparan sulfate in many tissues [56] and a distinct border of bound FGF2 was found surrounding the luminal epithelia and myoepithelia, in a pattern characteristic of basement membranes (arrowheads, Fig. **2A**). *Ndst1* ^f/f^ *MMTVCre* ^+^ mammary ducts displayed reduced binding of FGF2 in the basement membrane, while no change was observed in the staining of the mammary fat pad heparan sulfate (open arrowheads), consistent with the selective expression of Cre in the epithelial lineage. Mammary glands isolated from the *Ndst2* ^−/−^ animals resembled wildtype (data not shown), which is consistent with the lack of effect of Ndst2-deficiency on the heparan sulfate composition in most tissues [57].

Heparan sulfate was also analyzed chemically utilizing primary mammary epithelial cells isolated from multiparous *Ndst1* ^f/f^ *MMTVCre* ^+^ and *Ndst1* ^f/f^ *MMTVCre* ^–^ females. In these experiments, cells were labeled with [6-^3^H]glucosamine in culture, and labeled heparan sulfate was purified and the backbone of the polymer was cleaved at each N-sulfated glucosamine unit by treatment with nitrous acid at low pH [58]. The resulting oligosaccharides were then separated by gel filtration chromatography (Fig. **2B**). Wildtype heparan sulfate was typically rich in N-sulfated glucosamine residues located in adjacent disaccharides (dp2) or separated by one or two N-acetylated disaccharides (dp4 and dp6, respectively). In contrast, heparan sulfate produced by the mutant cells contained fewer of these smaller oligosaccharides and more extended structures rich in N-acetylated disaccharides (dp>18) were present. The areas under the peaks can be used to estimate the extent of N-sulfation of the chains [59]. Using this technique, heparan sulfate synthesized in the primary cells derived from *Ndst1* ^f/f^ *MMTVCre* ^+^ cells displayed a ∼2-fold reduction in N-sulfation (∼45% GlcNS in *Ndst1* ^f/f^ *MMTVCre* ^–^ cells *vs.* ∼25% in *Ndst1* ^f/f^ *MMTVCre* ^+^ cells) (Fig. 2C, inset). This decrease in GlcNAc N-deacetylation/N-sulfation is comparable to effects seen in mutant CHO cells lacking *Ndst1* [59] and in tissues derived from mice bearing a systemic null allele of *Ndst1* [41], [57], [60]. The FGF2 binding data (Fig. 2A) and the chemical analysis (Figs. 2B) indicate that *Ndst1* deficiency reduces the overall level of N-sulfation, which in turn can affect binding of heparin-binding growth factors of which FGF2 is a prototypical example.

### Normal branching morphogenesis but defective lobuloalveolar development in absence of *Ndst1*

**Figure 3:**
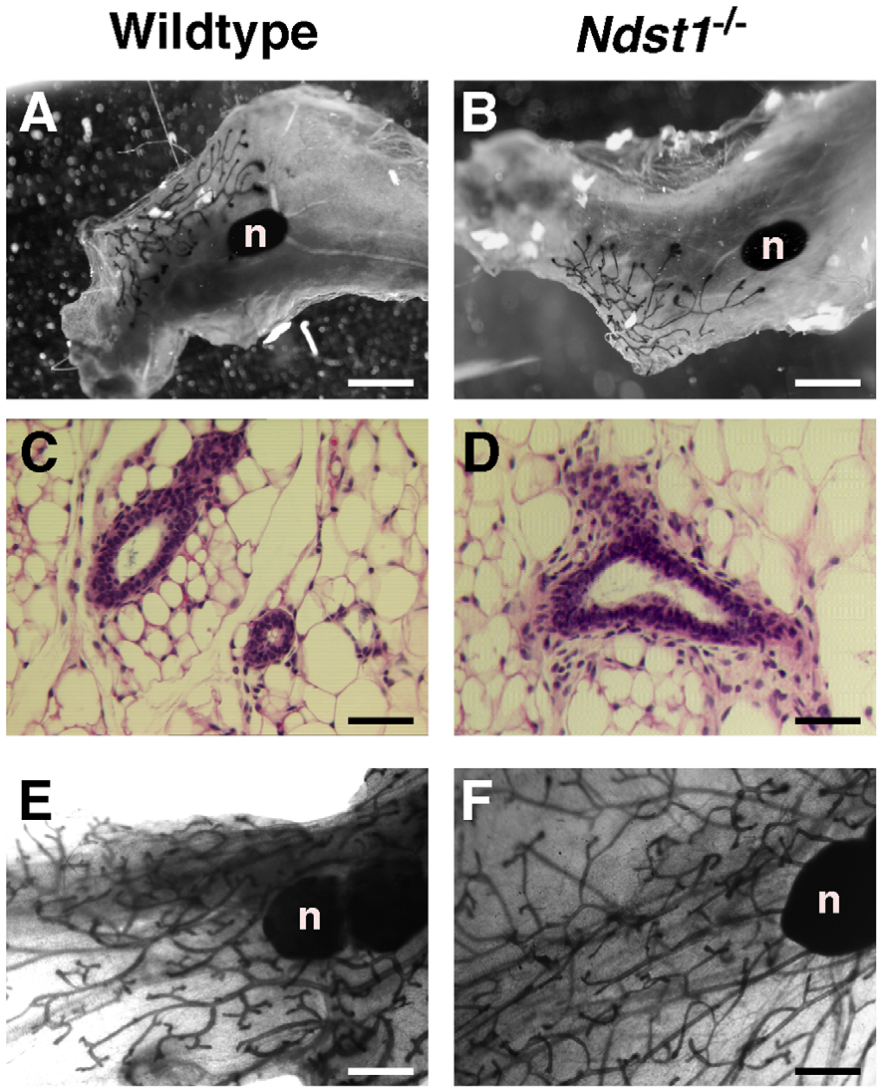
Branching morphogenesis is normal in NDST deficient epithelial cells. Whole mounts of the inguinal mammary glands were used to examine branching morphogenesis of one-month old *Ndst1* ^f/f^ *MMTVCre* ^–^ (Wildtype, **A**), and *Ndst1* ^f/f^ *MMTVCre* ^+^ (*Ndst1* ^–/–^, **B**) females. Deficiency of *Ndst1* has no effect on branching morphogenesis. Histological analysis of glands from one-month old animals showed normal organization of ductal epithelium (**C,D**). Whole mounts of 3 month-old glands also show normal branching (**E,F**). Bar  = 1.5 mm in the upper panels, 100 µm in the middle panels, and 1 mm in the lower panels. n, lymph node.

In wildtype mice, branching morphogenesis within the mammary gland occurs from birth to sexual maturity. By one month of age extensive branching has taken place, but the glands have not filled completely. Whole mount glands from one-month old Ndst1^−/−^ mice appeared normal with respect to the extent of fat pad colonization, overall branching, and the presence of terminal end buds (Fig. **3A,B**). Histological examination of hematoxylin/eosin-stained sections showed similar ductal density and similar gross arrangement of the surrounding connective tissue cells (Fig. **3C,D**). Comparable results were observed in 3-month old sexually mature animals as well, when the fat pad is filled completely and branching has ceased (Fig. **3E,F**). Numerous glands from animals randomized with respect to the estrus cycle were examined without any consistent differences in the glands. Thus, altering Ndst1 expression in glands had minimal effects on branching morphogenesis.

Ndst1-deficient mice gave birth to normal size litters, but less than 30% of the offspring from Ndst1-deficient females survived more than ∼1.5 days postpartum and the surviving pups were small. Fostering the pups with lactating female ICR mice allowed for normal development of pups, whereas ICR pups fostered to the *Ndst1* ^f/f^ *MMTVCre* ^+^ females perished shortly after the transfer. Necropsy revealed empty stomachs, suggesting that the Ndst1-deficient mothers did not properly lactate. Ndst2-deficient females yielded pups that thrived normally.

**Figure 4:**
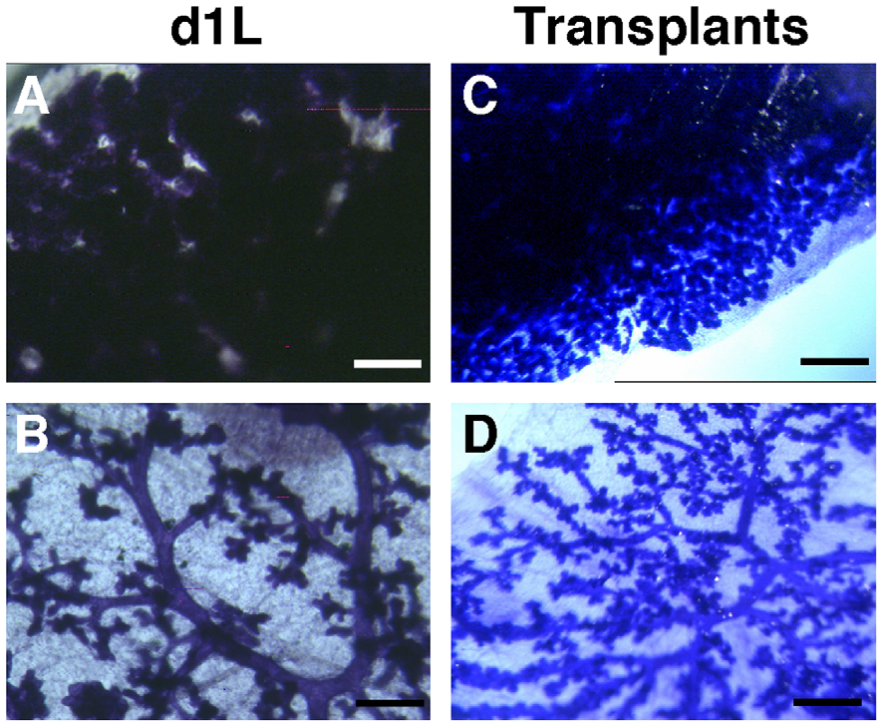
*Ndst1* ^f/f^ *MMTVCre* ^+^ mammary glands do not form lobuloalveoli. Whole mounts of inguinal mammary glands were used to examine branching morphogenesis of *Ndst1* ^f/f^ *MMTVCre* ^–^ (**A**) and *Ndst1* ^f/f^ *MMTVCre* ^+^ (**C**) D1L glands. *Ndst1*-deficient glands are grossly underdeveloped. (**B**) Transplantation of *Ndst1* ^f/f^ *MMTVCre* ^−^ epithelia into a cleared wildtype fat pad led to normal development at day 1 of lactation. (**D**) Transplantation of *Ndst1* ^f/f^ *MMTVCre* ^+^ epithelia showed a defect in lobuloalveoli development. (**A,C**): Bar  = 100 µm; (**B, D**): Bar  = 150 µm. n, lymph node.

**Figure 5:**
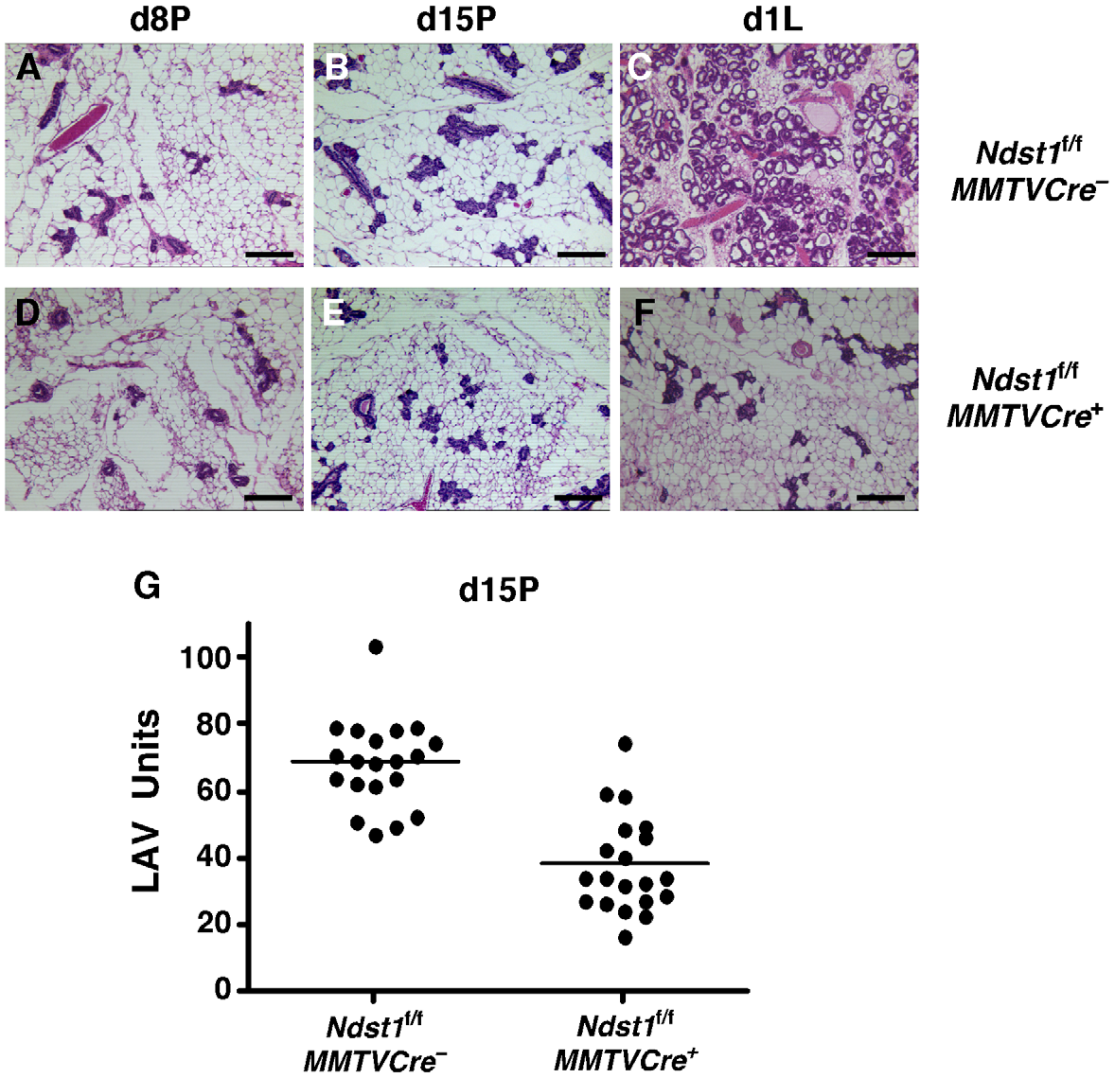
Hematoxylin/eosin stained sections of mammary glands. Mammary glands from D8P, D15P, and D1L *Ndst1* ^f/f^ *MMTVCre* ^−^ (**A–C, respectively**) and *Ndst1* ^f/f^ *MMTVCre* ^+^ (**D–F, respectively**) mice were stained with hematoxylin/eosin. No difference in glandular morphology was noted at D8P, but differences in ductal density occurred at D15P, which is quantified in (**G**). The difference in density between mutant and wildtype increased dramatically by D1L. Bars  = 125 µm.

Whole mounts of day 1 lactating (D1L) mammary glands from multiparous *Ndst1* ^f/f^ *MMTVCre* ^+^ females revealed dramatically retarded lobuloalveolar expansion compared to lactating glands from wildtype animals (compare Figs. **4A and 4B**), whereas whole mounts of multiparous non-pregnant *Ndst1* ^f/f^ *MMTVCre* ^+^ females looked normal (data not shown). Hematoxylin/eosin stained sections at day 1 of lactation indicated that the cellular architecture of the mammary ducts was normal, however the lobuloalveoli were reduced in size and number (compare Figs. **5C and 5F**). Analysis of stained sections of tissues obtained at earlier time points revealed that the disruption in lobuloalveolar development was not apparent at day 8 of pregnancy (compare Fig. **5A and 5D**), but was already manifest by day 15 of pregnancy (Fig. **5B and 5E**) where a slight reduction in lobuloalveolar expansion can be seen in the *Ndst1* ^f/f^ *MMTVCre* ^+^ females versus *Ndst1* ^ff^ *MMTVCre* ^−^ females. This reduction at d15P is quantified in Figure **5G**.

### Transplantation of *Ndst1* ^f/f^ *MMTVCre* ^+^ glands into wild-type mice

In order to confirm the epithelial autonomy of this phenotype, *Ndst1* ^f/f^ *MMTVCre* ^+^ epithelia were transplanted into the wild type fat pad cleared of epithelia from the inguinal gland. The host animals were bred two months later and whole mounts of the glands were analyzed at day 1 of lactation (D1L). Control wildtype fat pads receiving no epithelia remained devoid of ductal structures (data not shown), whereas those that received wildtype epithelia were normal (Fig. **4C**). Glands receiving *Ndst1* ^f/f^ *MMTVCre* ^+^ epithelia (Fig. **4D**) underwent ductal branching normally but exhibited the same reduced alveolar expansion seen in glands from D1L *Ndst1* ^f/f^ *MMTVCre* ^+^ females (Fig. **4B**). This finding confirmed that the lactation defect was due to the deletion of Ndst1 specifically in the mammary epithelia.

### Normal lactational differentiation in *Ndst1* ^f/f^ *MMTVCre* ^+^ mice

**Figure 6:**
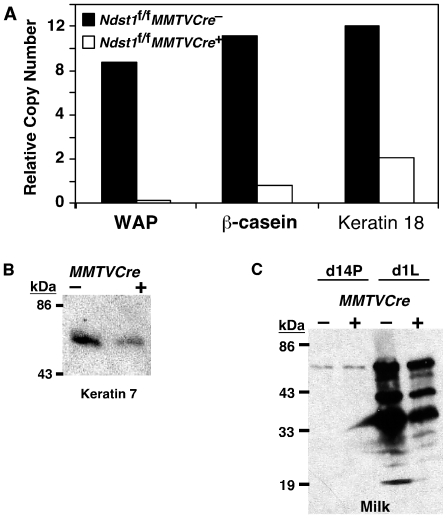
Characterization of the lactational defect in *Ndst1*-deficient mammary glands. Quantitative RT-PCR and Western blotting were used to characterize lactational capacity of *Ndst1* ^f/f^ *MMTVCre* ^+^ females. (**A**) RNA isolated from d1L *Ndst1* ^f/f^ *MMTVCre* ^–^ and *Ndst1* ^f/f^ *MMTVCre* ^+^ mammary glands was analyzed by quantitative RT-PCR for the expression of whey acidic protein (WAP), β-casein, and keratin 18, genes specifically expressed by mammary epithelial cells. Data was normalized to the expression of GAPDH transcripts present in the sample. (**B**) Western blotting with antibodies to keratin 7 in d1L glands further confirmed that the *Ndst1* ^f/f^ *MMTVCre* ^+^ mammary glands have a reduced population of epithelia. (**C**) Western blotting with antibodies to mouse milk from d1L glands shows that milk production was diminished in *Ndst1* ^f/f^ *MMTVCre* ^+^ glands. Blotting of d14P extracts showed that the antibodies were selective for milk protein except for a minor band at 67 kDa.

To further characterize the defect in the *Ndst1* ^f/f^ *MMTVCre* ^+^ females, casein and whey acidic protein (WAP) expression was analyzed. By quantitative PCR, the level of mRNAs of these proteins relative to GAPDH was dramatically reduced in d1L glands from *Ndst1* ^f/f^ *MMTVCre* ^+^ mice compared to *Ndst1* ^f/f^ *MMTVCre* ^–^ mice (Fig. **6A**). However, the decrease in milk protein expression paralleled changes in keratin 18 mRNA (Fig. **6A**) and keratin 7 protein expression (Fig. **6B**), markers for mammary epithelial cells, suggesting that the reduced capacity to produce milk might be related to the decrease in the number and size of lobuloalveoli rather than a defect in differentiation. Western blotting with antibodies to mouse milk from d1L glands showed that milk production was diminished in *Ndst1* ^f/f^ *MMTVCre* ^+^ glands nearly to the same extent as keratin expression (Fig. **6C**). Analysis of day 14 pregnant (d14P) glands showed that the antiserum was specific for milk proteins.

**Figure 7:**
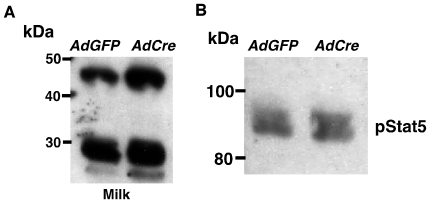
Normal differentiation in Ndst deficient epithelia. Mammary epithelia from *Ndst1^f/f^* animals were treated with AdGFP or AdCre and cultured on Matrigel in the presence of lactogenic hormones. The lactational response was measured by Western blotting with antibodies to milk proteins (**A**) and phosphorylated Stat5 (**B**). No differences were observed between AdGFP and AdCre infected cells. Milk protein induction or Stat5 phosphorylation was not detected without the addition of lactogenic hormones.

The aforementioned results suggested normal differentiation despite markedly decreased lobuloalveolar development. To confirm that differentiation was normal, primary mammary epithelial cells from mature *Ndst1* ^f/f^ females were plated on serum/fetuin coated plates and treated with adenoviral Cre (AdCre), which resulted in >95% deletion of *Ndst1* ^f/f^ allele. Passage of the cells in the presence of lactogenic hormones and Matrigel induced the epithelia to undergo differentiation into a lactational state in AdCre-treated cells and in cells infected with a control virus containing GFP (Fig. **7A**). Normal lactation has been shown to require signaling through the prolactin receptor, which activates milk protein gene transcription through the phosphorylation of Stat5. Western blotting with anti-phospho-Stat5 antibodies confirmed that proper signaling had occurred (Fig. **7B**). Taken together, the data show that differentiation was normal in Ndst1-deficient glands and that the paucity of milk was due to a defect in the proliferation of the epithelial population required for lactation.

## Discussion

Mouse mammary development proceeds through multiple morphologically well-defined steps. After birth, the epithelial placode undergoes branching morphogenesis from a rudimentary branched structure to a fully branched ductal tree that fills the fat pad. During pregnancy, further secondary and tertiary branching occurs and alveoli appear along the ducts, resulting eventually in the formation of lobuloalveoli that can differentiate into milk producing glands under the influence of lactogenic hormones. After weaning, the gland involutes by apoptosis of the epithelial cells, returning the gland to the virgin state. Each stage in this process depends on a series of growth factors, many of which can interact with heparan sulfate [23].

To examine if these interactions are necessary for post-natal mammary development, we reduced the level of sulfation of the chains by inactivating *Ndst1* using the Cre recombinase under the control of the MMTV promoter. At least 80% knockout was achieved in the in vivo experiments and >95% deletion was achieved in vitro. Because this system depends on hormonal activation during the estrous cycle, we cannot make any conclusions regarding the role of heparan sulfate on embryonic stages of differentiation. Given the large number of growth factors and interactions involved in branching morphogenesis, we were surprised that the alteration in sulfation did not result in failure of the gland to undergo branching morphogenesis. Although it is conceivable that deletion of the remaining 20% of Ndst1 gene could affect branching, transplantation studies with epithelia treated with AdCre also underwent branching in an apparently normal manner. In marked contrast to branching, altering heparan sulfate in this way had a striking and fully penetrant effect on lobuloalveolar expansion in female mice, leading to insufficient milk production to support their offspring (Fig. 6). Thus, this is the first report of a heparan sulfate gene modification that selectively blocks lobuloalveolar development while largely, if not completely, sparing branching morphogenesis.

Lobuloalveolar development depends on sophisticated coordination between proliferative and apoptotic signals. During lobuloalveolar expansion, local growth factors released from the surrounding fat pad induce quiescent epithelia to undergo proliferation, resulting in alveolar budding, lobuloalveolar development and milk production. Lactogenic differentiation proceeds normally in Ndst1-deficient glands and isolated epithelia (Fig. 7), suggesting a discrete effect on budding and lobuloalveoli formation. The focal defect on alveolar development presumably arose from inefficient growth factor action due to failure to form appropriate complexes of ligands, signaling receptors and heparan sulfate [23]. Relevant factors at this stage of development include one or more FGFs based on partial inhibition of lobuloalveoli in animals bearing a dominant-negative FGFR2IIIb construct [61]. Although markedly reduced binding of FGF2 was observed in the basement membrane of mutant gland epithelia (but not the fat pad adipocytes, which had FGF2 binding similar to controls, Fig. **2**), the relevant FGF ligands active at this stage have yet to be identified [15], [62], [63]. Mutants altered in HRGα expression and ErbB4 receptors also show reduced lobuloalveolar development [12]. Since both FGF signaling and ErbB4 mediated signaling are regulated by heparan sulfate, further studies are needed to identify the specific growth factors actually undermined by Ndst1-deficiency in vivo. Conceivably, the alteration could also affect the differentiation of stem cell precursors that contribute to a specific niche in lobuloalveolar expansion [64], [65].

During weaning, the mammary gland undergoes involution, in which the secretory epithelium is destroyed and adipocyte proliferation fills the fat pad. The first stage is characterized by apoptosis of the epithelium, and multiple factors participate in activation of the death pathway, including leukemia inhibitory factor (LIF), insulin-like growth factors (IGF-I and IGF-II) and their binding proteins (IGFBP-5), wnt and TGF-β [66]. Many of these proapoptotic factors bind to heparan sulfate. Thus, altering the structure of heparan sulfate by inactivation of *Ndst1* could inhibit multiple steps in the cascade, which may explain the highly penetrant defect in Ndst1-deficient mice.

It seems likely that these studies have implications for other organ systems that undergo branching morphogenesis, e.g. the salivary gland [67], lung [68], kidney [47], [48], [69], prostate gland [70], and lacrimal gland [71]. Certain growth factor-heparan sulfate interactions have been proposed to act as “switches” during the stages of kidney organogenesis [69]. Many of these systems appear to depend on heparan sulfate and the array of growth factors implicated in branching overlaps considerably with those involved in mammary gland development. Therefore, one might predict that altering sulfation by silencing specific sulfotransferases in these organs could tease out different growth factor-heparan sulfate interactions required during different stages of development in these organs.

## Materials and Methods

### Ethics Statement

All animals were handled in accordance with protocols for the humane treatment of animals. This study was approved by the IACUC and Animal Subjects Committee at the University of California, San Diego (Protocol S99127).

### Transgenic mice

Mice bearing a loxP-flanked allele of *Ndst1* were described previously [41]. The MMTV Cre line “A” mice in the 129 background was obtained from Dr. T. Wynshaw-Boris (University of California, San Diego) [53], [54]. Only male mice carrying the MMTV Cre allele were used for breeding to avoid deletion of the of the conditional allele by Cre expression in oocytes. All experiments were done with mice on a mixed background with littermate controls. Qualitative and quantitative aspects of phenotype did not change with further backcrossing of *Ndst1* ^f/f^ into the C57Bl/6 background.

### Cell culture

Primary mammary epithelia were isolated and cultured following an established protocol [72]. Briefly, number 4 and 5 glands were excised and chopped with a razor blade, digested with 0.2% trypsin and 0.2% collagenase A and cells and organoids were enriched by differential centrifugation. Multiwell tissue culture plates were precoated with 100 µl/cm^2^ of Ham's F12 medium containing 20% heat-inactivated fetal bovine serum (FBS) and 1 mg/ml fetuin. Cells were cultured in Ham's F12 medium containing 10% heat-inactivated FBS, 5 µg/ml insulin, 1 µg/ml hydrocortisone, 5 ng/ml epidermal growth factor, 50 µg/ml gentamycin, 100 U/ml penicillin, and 100 µg/ml streptomycin. The medium was changed every two days.

Lactational differentiation was induced by established methods [73]. Briefly, first passage mammary epithelia were plated on serum/fetuin coated plates with Matrigel and cultured for 4 days in DMEM/F12 media containing 5 µg/ml insulin, 1 µg/ml hydrocortisone, 3 µg/ml prolactin, 50 µg/ml gentamycin, 100 U/ml penicillin, and 100 µg/ml streptomycin.

An adenovirus containing Cre recombinase (AdCre) was used to inactivate *Ndst1* in vitro, as described [74]. AdCre and adenovirus containing green fluorescent protein (AdGFP) were obtained from the Vector Core Development Lab at the University of California, San Diego. Cells were treated twice over four days with 10^8^ pfu/ml media for 90 minutes, washed with PBS and cultured in normal growth medium. Flow cytometry using biotinylated FGF and streptavidin phycoerythrin-Cy5 showed >10-fold shift in fluorescence of ∼99% of the cells [75].

### Mammary gland histology

Histological analyses were performed by the Cancer Center Histology Core at the University of California, San Diego. Whole mounts were stained with hematoxylin. Binding of FGF2 to heparan sulfate was measured in frozen sections of the inguinal mammary gland using biotinylated FGF2 and HRP-streptavidin [60]. Hematoxylin and eosin staining of sections was performed by standard procedures.

### Southern blotting and RT-PCR analysis

DNA was isolated from primary mammary epithelial from mice of various ages using the Qiagen DNeasy tissue kit (Qiagen, Valencia, CA). DNA (20 µg) was digested with Hind III and Bgl II overnight and analyzed by agarose gel (0.8%) electrophoresis. After transfer to a nylon membrane, the blot was probed with a genomic probe located outside of the deleted exon [41].

RNA was isolated from purified mammary epithelial cells (TRIzol reagent), reverse transcribed (Superscript III; Invitrogen, Carlsbad, CA) and amplified using gene specific primers to each Ndst isoform (*Ndst1*, Forward: GGACATCTGGTCTAAG, Reverse: GATGCCTTTGTGATAG; *Ndst2*, Forward: GATGACAAGAGGCAC, Reverse: CAGTGCTGGCATTGG; *Ndst3*, Forward: CCACTGCCTTGTGTC, Reverse: GGAGTACGCTCGGTC; *Ndst4*, Forward: CTAACTACTTCCACTC, Reverse: ATGTGCACTGCATACC).

Q-PCR analysis of β-casein, whey acidic protein and keratin18 was done by the Genomics Core Facility at the University of California San Diego. The following primers were used: β-casein, Forward: AGGTGAATCTCATGGGACAGCT and Reverse: TGACTGGATGCTGGAGTGAACT; whey acidic protein, Forward: TGCCATGTGCTGTCCCG and Reverse: CCAGCTTTCGGAACACCAAT; keratin 18, Forward: CAGTATGAAGCGCTGGCTCA and Reverse: GTGGTACTCTCCTCAATCTGCTGA.

### Western blotting

Whole gland protein was prepared by removing the lymph node from the number 4 mammary gland and homogenization [76]. Protein content was determined with the Bradford assay using BSA as a standard (BioRad, Hercules, CA). Ten micrograms of protein from whole glands or cultured cells was electrophoresed on BioRad precast Ready gels and transferred to nitrocellulose with a semi-dry blotting apparatus. The following antibodies were used: anti-mouse milk antisera (Nordic Immunological Laboratories, Tilburg, The Netherlands); pStat5 (Tyr694), and keratan-7. HRP-conjugated anti-mouse and anti-goat IgG were obtained from BioRad (Hercules, CA). HRP was detected using the SuperSignal West Pico chemiluminescent substrate (Pierce, Rockford, Il).

### Heparan sulfate analysis

Subconfluent primary mammary epithelial cells were cultured for 48 hours in DMEM containing 2 mM glucose, 10% dialyzed FBS, 5 µg/ml insulin, 1 µg/ml hydrocortisone, 5 ng/ml epidermal growth factor and 50 µCi/ml [6-^3^H]glucosamine (New England Nuclear, Waltham, MA). Cell associated and secreted glycosaminoglycans were isolated, cleaved with nitrous acid pH 1.5 [58], and fractionated by gel filtration chromatography as described [59].

## References

[1] Hovey RC, Trott JF, Vonderhaar BK (2002). Establishing a framework for the functional mammary gland: from endocrinology to morphology.. J Mammary Gland Biol Neoplasia.

[2] Brisken C, Ayyannan A, Nguyen C, Heineman A, Reinhardt F (2002). IGF-2 is a mediator of prolactin-induced morphogenesis in the breast.. Dev Cell.

[3] Brisken C, Heineman A, Chavarria T, Elenbaas B, Tan J (2000). Essential function of Wnt-4 in mammary gland development downstream of progesterone signaling.. Genes Dev.

[4] Chapman RS, Lourenco PC, Tonner E, Flint DJ, Selbert S (1999). Suppression of epithelial apoptosis and delayed mammary gland involution in mice with a conditional knockout of Stat3.. Genes Dev.

[5] Dunbar ME, Dann P, Brown CW, Van Houton J, Dreyer B (2001). Temporally regulated overexpression of parathyroid hormone-related protein in the mammary gland reveals distinct fetal and pubertal phenotypes.. J Endocrinol.

[6] Forrester E, Chytil A, Bierie B, Aakre M, Gorska AE (2005). Effect of conditional knockout of the type II TGF-beta receptor gene in mammary epithelia on mammary gland development and polyomavirus middle T antigen induced tumor formation and metastasis.. Cancer Res.

[7] Grimm SL, Seagroves TN, Kabotyanski EB, Hovey RC, Vonderhaar BK (2002). Disruption of steroid and prolactin receptor patterning in the mammary gland correlates with a block in lobuloalveolar development.. Mol Endocrinol.

[8] Humphreys RC, Lydon J, O'Malley BW, Rosen JM (1997). Mammary gland development is mediated by both stromal and epithelial progesterone receptors.. Mol Endocrinol.

[9] Joseph H, Gorska AE, Sohn P, Moses HL, Serra R (1999). Overexpression of a kinase-deficient transforming growth factor-beta type II receptor in mouse mammary stroma results in increased epithelial branching.. Mol Biol Cell.

[10] Lewis MT, Ross S, Strickland PA, Sugnet CW, Jimenez E (2001). The Gli2 transcription factor is required for normal mouse mammary gland development.. Dev Biol.

[11] Lewis MT, Ross S, Strickland PA, Sugnet CW, Jimenez E (1999). Defects in mouse mammary gland development caused by conditional haploinsufficiency of Patched-1.. Development.

[12] Li L, Cleary S, Mandarano MA, Long W, Birchmeier C (2002). The breast proto-oncogene, HRGalpha regulates epithelial proliferation and lobuloalveolar development in the mouse mammary gland.. Oncogene.

[13] Long W, Wagner KU, Lloyd KC, Binart N, Shillingford JM (2003). Impaired differentiation and lactational failure of Erbb4-deficient mammary glands identify ERBB4 as an obligate mediator of STAT5.. Development.

[14] Luetteke NC, Qiu TH, Fenton SE, Troyer KL, Riedel RF (1999). Targeted inactivation of the EGF and amphiregulin genes reveals distinct roles for EGF receptor ligands in mouse mammary gland development.. Development.

[15] Mailleux AA, Spencer-Dene B, Dillon C, Ndiaye D, Savona-Baron C (2002). Role of FGF10/FGFR2b signaling during mammary gland development in the mouse embryo.. Development.

[16] Robinson GW, Hennighausen L (1997). Inhibins and activins regulate mammary epithelial cell differentiation through mesenchymal-epithelial interactions.. Development.

[17] Ruan W, Kleinberg DL (1999). Insulin-like growth factor I is essential for terminal end bud formation and ductal morphogenesis during mammary development.. Endocrinology.

[18] Tidcombe H, Jackson-Fisher A, Mathers K, Stern DF, Gassmann M (2003). Neural and mammary gland defects in ErbB4 knockout mice genetically rescued from embryonic lethality.. Proc Natl Acad Sci USA.

[19] Wiesen JF, Young P, Werb Z, Cunha GR (1999). Signaling through the stromal epidermal growth factor receptor is necessary for mammary ductal development.. Development.

[20] Xie W, Paterson AJ, Chin E, Nabell LM, Kudlow JE (1997). Targeted expression of a dominant negative epidermal growth factor receptor in the mammary gland of transgenic mice inhibits pubertal mammary duct development.. Mol Endocrinol.

[21] Yang Y, Spitzer E, Meyer D, Sachs M, Niemann C (1995). Sequential requirement of hepatocyte growth factor and neuregulin in the morphogenesis and differentiation of the mammary gland.. J Cell Biol.

[22] Bishop JR, Schuksz M, Esko JD (2007). Heparan sulphate proteoglycans fine-tune mammalian physiology.. Nature.

[23] Delehedde M, Lyon M, Sergeant N, Rahmoune H, Fernig DG (2001). Proteoglycans: pericellular and cell surface multireceptors external stimuli in the mammary gland.. J Mammary Gland Biol Neoplasia.

[24] Lander AD, Nie Q, Wan FY (2002). Do morphogen gradients arise by diffusion?. Dev Cell.

[25] Yu WH, Woessner JF (2000). Heparan sulfate proteoglycans as extracellular docking molecules for matrilysin (matrix metalloproteinase 7).. J Biol Chem.

[26] Gordon JR, Bernfield MR (1980). The basal lamina of the postnatal mammary epithelium contains glycosaminoglycans in a precise ultrastructural organization.. Dev Biol.

[27] Silberstein GB, Daniel CW (1982). Glycosaminoglycans in the basal lamina and extracellular matrix of the developing mouse mammary duct.. Dev Biol.

[28] Zcharia E, Metzger S, Chajek-Shaul T, Aingorn H, Elkin M (2004). Transgenic expression of mammalian heparanase uncovers physiological functions of heparan sulfate in tissue morphogenesis, vascularization, and feeding behavior.. Faseb J.

[29] Alexander CM, Reichsman F, Hinkes MT, Lincecum J, Becker KA (2000). Syndecan-1 is required for Wnt-1-induced mammary tumorigenesis in mice.. Nat Genet.

[30] Liu BY, Kim YC, Leatherberry V, Cowin P, Alexander CM (2003). Mammary gland development requires syndecan-1 to create a beta-catenin/TCF-responsive mammary epithelial subpopulation.. Oncogene.

[31] Yu WH, Woessner JF, McNeish JD, Stamenkovic I (2002). CD44 anchors the assembly of matrilysin/MMP-7 with heparin-binding epidermal growth factor precursor and ErbB4 and regulates female reproductive organ remodeling.. Genes Dev.

[32] Arikawa-Hirasawa E, Watanabe H, Takami H, Hassell JR, Yamada Y (1999). Perlecan is essential for cartilage and cephalic development.. Nat Genet.

[33] Cano-Gauci DF, Song HH, Yang H, McKerlie C, Choo B (1999). Glypican-3-deficient mice exhibit developmental overgrowth and some of the abnormalities typical of Simpson-Golabi-Behmel syndrome.. JCell Biol.

[34] Echtermeyer F, Streit M, Wilcox-Adelman S, Saoncella S, Denhez F (2001). Delayed wound repair and impaired angiogenesis in mice lacking syndecan-4.. J Clin Invest.

[35] Ishiguro K, Kadomatsu K, Kojima T, Muramatsu H, Matsuo S (2001). Syndecan-4 deficiency increases susceptibility to kappa-carrageenan-induced renal damage.. Lab Invest.

[36] Ishiguro K, Kadomatsu K, Kojima T, Muramatsu H, Iwase M (2001). Syndecan-4 deficiency leads to high mortality of lipopolysaccharide-injected mice.. J Biol Chem.

[37] Zhou Z, Wang J, Cao R, Morita H, Soininen R (2004). Impaired angiogenesis, delayed wound healing and retarded tumor growth in perlecan heparan sulfate-deficient mice.. Cancer Res.

[38] Lin X, Wei G, Shi ZZ, Dryer L, Esko JD (2000). Disruption of gastrulation and heparan sulfate biosynthesis in EXT1-deficient mice.. Dev Biol.

[39] Stickens D, Zak BM, Rougier N, Esko JD, Werb Z (2005). Mice deficient in Ext2 lack heparan sulfate and develop exostoses.. Development.

[40] Fan G, Xiao L, Cheng L, Wang X, Sun B (2000). Targeted disruption of NDST-1 gene leads to pulmonary hypoplasia and neonatal respiratory distress in mice.. FEBS Lett.

[41] Grobe K, Inatani M, Pallerla SR, Castagnola J, Yamaguchi Y (2005). Cerebral hypoplasia and craniofacial defects in mice lacking heparan sulfate Ndst1 gene function.. Development.

[42] Ringvall M, Ledin J, Holmborn K, Van Kuppevelt T, Ellin F (2000). Defective heparan sulfate biosynthesis and neonatal lethality in mice lacking N-deacetylase/N-sulfotransferase-1.. J Biol Chem.

[43] Bullock SL, Fletcher JM, Beddington RS, Wilson VA (1998). Renal agenesis in mice homozygous for a gene trap mutation in the gene encoding heparan sulfate 2-sulfotransferase.. Genes Dev.

[44] Li JP, Gong F, Hagner-McWhirter A, Forsberg E, Abrink M (2003). Targeted disruption of a murine glucuronyl C5-epimerase gene results in heparan sulfate lacking L-iduronic acid and in neonatal lethality.. J Biol Chem.

[45] Merry CLR, Bullock SL, Swan DC, Backen AC, Lyon M (2001). The molecular phenotype of heparan sulfate in the *Hs2st*
^-/-^ mutant mouse.. J Biol Chem.

[46] Stanford KI, Wang L, Castagnola J, Song D, Bishop JR (2010). Heparan sulfate 2-O-sulfotransferase is required for triglyceride-rich lipoprotein clearance.. J Biol Chem.

[47] Steer DL, Shah MM, Bush KT, Stuart RO, Sampogna RV (2004). Regulation of ureteric bud branching morphogenesis by sulfated proteoglycans in the developing kidney.. Dev Biol.

[48] Shah MM, Sakurai H, Sweeney DE, Gallegos TF, Bush KT (2010). Hs2st mediated kidney mesenchyme induction regulates early ureteric bud branching.. Dev Biol.

[49] Esko JD, Selleck SB (2002). Order out of chaos: Assembly of ligand binding sites in heparan sulfate.. Annu Rev Biochem.

[50] Grobe K, Ledin J, Ringvall M, Holmborn K, Forsberg E (2002). Heparan sulfate and development: Differential roles of the N-acetylglucosamine N-deacetylase/N-sulfotransferase isozymes.. Biochim Biophys Acta Gen Subj.

[51] Forsberg E, Pejler G, Ringvall M, Lunderius C, Tomasini-Johansson B (1999). Abnormal mast cells in mice deficient in a heparin-synthesizing enzyme.. Nature.

[52] Humphries DE, Wong GW, Friend DS, Gurish MF, Qiu WT (1999). Heparin is essential for the storage of specific granule proteases in mast cells.. Nature.

[53] Wagner KU, Wall RJ, St-Onge L, Gruss P, Wynshaw-Boris A (1997). Cre-mediated gene deletion in the mammary gland.. Nucleic Acids Res 25.

[54] Wagner KU, McAllister K, Ward T, Davis B, Wiseman R (2001). Spatial and temporal expression of the Cre gene under the control of the MMTV-LTR in different lines of transgenic mice.. Transgenic Res.

[55] Bai XM, Wei G, Sinha A, Esko JD (1999). Chinese hamster ovary cell mutants defective in glycosaminoglycan assembly and glucuronosyltransferase I.. J Biol Chem.

[56] Allen BL, Rapraeger AC (2003). Spatial and temporal expression of heparan sulfate in mouse development regulates FGF and FGF receptor assembly.. J Cell Biol.

[57] Ledin J, Staatz W, Li JP, Gotte M, Selleck S (2004). Heparan sulfate structure in mice with genetically modified heparan sulfate production.. J Biol Chem.

[58] Shively JE, Conrad HE (1976). Formation of anhydrosugars in the chemical depolymerization of heparin.. Biochemistry.

[59] Bame KJ, Esko JD (1989). Undersulfated heparan sulfate in a Chinese hamster ovary cell mutant defective in heparan sulfate N-sulfotransferase.. J Biol Chem.

[60] Wang L, Fuster M, Sriramarao P, Esko JD (2005). Endothelial heparan sulfate deficiency impairs L-selectin- and chemokine-mediated neutrophil trafficking during inflammatory responses.. Nat Immunol.

[61] Jackson D, Bresnick J, Rosewell I, Crafton T, Poulsom R (1997). Fibroblast growth factor receptor signalling has a role in lobuloalveolar development of the mammary gland.. J Cell Sci.

[62] Dono R, Texido G, Dussel R, Ehmke H, Zeller R (1998). Impaired cerebral cortex development and blood pressure regulation in FGF-2-deficient mice.. Embo J.

[63] Ortega S, Ittmann M, Tsang SH, Ehrlich M, Basilico C (1998). Neuronal defects and delayed wound healing in mice lacking fibroblast growth factor 2.. Proc Natl Acad Sci USA.

[64] Paguirigan A, Beebe DJ, Liu B, Alexander C (2006). Mammary stem and progenitor cells: tumour precursors?. Eur J Cancer.

[65] Stingl J, Eirew P, Ricketson I, Shackleton M, Vaillant F (2006). Purification and unique properties of mammary epithelial stem cells.. Nature.

[66] Green KA, Streuli CH (2004). Apoptosis regulation in the mammary gland.. Cell Mol Life Sci.

[67] Patel VN, Rebustini IT, Hoffman MP (2006). Salivary gland branching morphogenesis.. Differentiation.

[68] Cardoso WV, Lu J (2006). Regulation of early lung morphogenesis: questions, facts and controversies.. Development.

[69] Shah MM, Sampogna RV, Sakurai H, Bush KT, Nigam SK (2004). Branching morphogenesis and kidney disease.. Development.

[70] Thomson AA, Marker PC (2006). Branching morphogenesis in the prostate gland and seminal vesicles.. Differentiation.

[71] Pan Y, Carbe C, Powers A, Zhang EE, Esko JD (2008). Bud specific N-sulfation of heparan sulfate regulates Shp2-dependent FGF signaling during lacrimal gland induction.. Development.

[72] Pullan SE, Streuli CH, Harris A (1996). The mammary gland epithelial cell.. Epithelial Cell Culture: Cambridge.

[73] Streuli CH, Edwards GM, Delcommenne M, Whitelaw CB, Burdon TG (1995). Stat5 as a target for regulation by extracellular matrix.. J Biol Chem.

[74] Li M, Wagner KU, Furth PA, Asch BB (2000). Transfection of primary mammary epithelial cells by viral and nonviral methods.. Methods in Mammary Gland Biology and Breast Cancer Research.

[75] Wei G, Bai XM, Gabb MMG, Bame KJ, Koshy TI (2000). Location of the glucuronosyltransferase domain in the heparan sulfate copolymerase EXT1 by analysis of Chinese hamster ovary cell mutants.. J Biol Chem.

[76] Faraldo MM, Deugnier MA, Thiery JP, Glukhova MA (2001). Growth defects induced by perturbation of beta1-integrin function in the mammary gland epithelium result from a lack of MAPK activation via the Shc and Akt pathways.. EMBO Rep.

